# The supplementation of the multi-strain probiotics WHHPRO™ alleviates high-fat diet-induced metabolic symptoms in rats via gut-liver axis

**DOI:** 10.3389/fnut.2023.1324691

**Published:** 2024-01-11

**Authors:** Cailing Chen, Kan Gao, Zuoguo Chen, Qiwen Zhang, Xueqin Ke, Bingyong Mao, Qiuling Fan, Yanjun Li, Su Chen

**Affiliations:** ^1^Key Laboratory of Food and Biological Engineering of Zhejiang Province, Hangzhou, China; ^2^Research and Development Department, Hangzhou Wahaha Group Co., Ltd., Hangzhou, China; ^3^Hangzhou Wahaha Technology Co., Ltd., Hangzhou, China; ^4^School of Food Science and Technology, Jiangnan University, Wuxi, China

**Keywords:** WHHPRO, gut-liver axis, bile acid, SCFA, metabolic syndrome

## Abstract

Metabolic syndrome (MS) has emerged as one of the major global health concerns, accompanied by a series of related complications, such as obesity and type-2 diabetes. The gut-liver axis (GLA) is a bidirectional communication between the gut and the liver. The GLA alterations have been revealed to be closely associated with the development of MS. Probiotics within *Lactobacillus* and *Bifidobacterium* confer beneficial effects on improving MS symptoms. WHHPRO™ is a mixture of four probiotic strains, with potential MS-improving abilities. This study aimed to investigate the effects of WHHPRO™ on MS symptoms using a high-fat diet (HFD) rat model. Oral administration of WHHPRO™ for 12 weeks improved glucose tolerance, blood lipid, body weight, and liver index in HFD rats. WHHPRO™ shaped the gut microbiome composition by increasing the abundance of *Lactobacillus* and *Akkermansia* and normalized the reduced SCFA levels in HFD rats. Besides, WHHPRO™ modulated the fecal bile acids (BAs) profile, with decreased levels of T-b-MCA and 12-KDCA and increased levels of LCA and ILCA. Meanwhile, WHHPRO™ increased total unconjugated BAs in feces and liver and reduced the accumulation of total hepatic BA pool size in HFD rats. Moreover, WHHPRO™ reversed the expression of genes associated with impaired BA metabolism signaling in the ileum and liver. Our findings suggest that WHHPRO™ exerted beneficial effects on improving MS symptoms, involving the modulation of the gut microbiome composition, SCFAs, and the FXR-FGF15 signaling along the GLA. Supplementation of WHHPRO™ may serve as a novel strategy for improving MS symptoms.

## Introduction

Metabolic syndrome (MS) is a complex and multi-factorial medical condition that encompasses a constellation of disorders, including insulin resistance, glucose intolerance, hyperglycemia, hyperlipidemia, hypertension, and abdominal obesity (1). The International Diabetes Federation (IDF) has proposed definitive diagnostic criteria for MS based on the presence of at least three of the following five parameters: obesity raised fasting blood glucose (FBG), raised blood pressure, raised triacylglycerol (TG), and reduced high-density lipoprotein cholesterol (HDL-C) (2). Despite the availability of pharmaceutical interventions for MS, these treatments often have unsatisfactory outcomes and can be associated with side effects such as gastrointestinal discomfort and hepatic injury (3). Therefore, the development of therapeutic strategies that are safe, effective, and well-tolerated is urgently needed.

The gastrointestinal (GI) tract is a complex anaerobic bioreactor populated by a multitude of microorganisms that play a crucial role in maintaining host health. The human gut microbiota is dominated by four bacterial phyla, namely Bacteroidetes, Firmicutes, Proteobacteria, and Actinobacteriota, which collectively constitute more than 99% of the gut bacterial population (4). The gut microbiota plays an essential role in the development and maintenance of host health, and disturbances of the gut microbiota have been associated with metabolic disorders including MS (5, 6). Therefore, modulating the composition of the gut microbiome represents a promising approach for the treatment of MS and associated metabolic disorders.

Alterations in the composition of the gut microbiome were accompanied by changes in the metabolism of short-chain fatty acids (SCFAs) and bile acids (BAs) (7, 8). SCFAs have been shown to play a critical role in maintaining intestinal homeostasis and modulating host metabolism, leading to improved intestinal barrier function, shaped immune responses, and enhanced energy metabolism (8). Concurrently, high concentrations of SCFAs in the gut can lower the pH, thus inhibiting the growth of potential pathogens, and promoting the growth of beneficial bacteria such as *Bifidobacterium* and *Lactobacillus*, which are well-known microorganisms associated with the attenuating risk factors for various diseases (9, 10).

The gut-liver axis (GLA) refers to the bidirectional communication and interaction between the gut and the liver, given that the liver receives approximately 70% of its blood supply from the intestine and is exposed to intestinal-derived metabolites (11). Intestinal signaling is known to play a critical role in the pathogenesis of liver disease, particularly in the circulation of bile acids along the GLA (12, 13). BAs are synthesized in hepatocytes and then secreted into the small intestine. BAs serve as essential digestive surfactants, aiding in the emulsification of dietary lipids and facilitating their absorption, thereby promoting the digestion and uptake of fats. Approximately 95% of BAs in the gut are absorbed by the intestinal wall and returned to the liver via the portal vein (10).

A portion of BAs can be metabolized by gut microbiota, forming metabolites that act as signaling molecules and participate in the regulation of physiological processes in the liver and gut (14). The classical pathway, modulated by the rate-limiting enzyme cholesterol 7*a*-hydroxylase (CYP7A1), accounts for approximately 90% of BA production (10, 11). Additionally, in the intestine, specific BAs can modulate the activity of farnesoid X receptor (FXR), inducing the gene expressions of *small heterodimer partner* (*Shp*) and *fibroblast growth factor 15* (*Fgf15*) in mice or *Fgf19* in humans, ultimately, inhibiting the gene expressions of *Cyp7a1*, thereby leading to a reduction in BA production (10–12, 15). Previous studies have revealed that alterations in the GLA are closely associated with the development of MS, with changes in the levels of BAs and the gene expressions of *Cyp7a1*, *Fxr*, and *Fgf15* (16, 17). These findings point to the potential role of GLA in the treatment of liver-gut-related diseases.

Probiotics, including the probiotic *Lactobacillus*, one of the most beneficial bacteria in the gastrointestinal tract of mammals, are beneficial live microorganisms that confer health benefits to the host and have received considerable attention due to their potential beneficial effects on various diseases, including MS (18). Probiotics exert their effects through multiple mechanisms, the most important of which is to modulate the structure of the gut microbial community and relevant metabolites, thereby influencing metabolic functions (6, 7, 19). Therefore, probiotic supplementation may be one of the possible treatment strategies to improve MS symptoms. However, the underlying mechanism by which probiotics confer these beneficial effects on improving MS remains unclear.

Evidence has suggested that certain probiotic strains, such as *Bifidobacterium animalis*, *Lactobacillus rhamnosus*, and *Limosilactobacillus fermentum* (20, 21), may exert beneficial effects in attenuating the risk factors associated with MS. Accordingly, a multi-strain probiotic mixture, designated as WHHPRO™, has been developed, consisting of *B. animalis* WHH2276, *L. rhamnosus* WHH1155, *L. fermentum* WHH2644, and WHH3906, with each strain exhibiting MS-improving potentials. Therefore, the present study aimed to investigate the MS-improving effects of WHHPRO™ in an HFD rat model, which has been commonly used as one of the classical models of MS (22). The changes in glucose tolerance, blood lipid, body weight, organ index, gut microbiome composition, fecal SCFAs, BA profile in the feces and liver, and FXR-FGF15 signaling gene expressions were investigated.

## Materials and methods

### The design of probiotic mixture WHHPRO™

WHHPRO™, a multi-strain probiotic mixture product, was designed and provided by Hangzhou Wahaha Group Co. Ltd (Hangzhou, China). The probiotic mixture consisted of four strains, *B. animalis* WHH2276, *L. rhamnosus* WHH1155, *L. fermentum* WHH2644, and WHH3906. All strains were preserved in the China General Microbiological Culture Collection Centre (CGMCC) under the identity numbers CGMCC 62901, 16754, 11955, and 19472. According to our pre-experiment in an MS zebrafish model (the experimental procedures are provided in Supplementary Information), each strain exhibited potential MS-improving abilities, with blood glucose, TG, and total cholesterol (TC)-lowing effects (Supplementary Figures 1–3). To enhance the MS-improving efficacy, four strains were combined and accompanied by prebiotics. The detailed composition of WHHPRO™ is presented in Supplementary Table 1.

### Animals, diets, and sampling

All animal experiments were conducted following the guidelines and regulations of the “Governing Regulation for the Use of Experimental Animals” in Zhejiang Province, China. The animal care and study protocols were specifically reviewed and approved by the Animal Care and Use Committee of Hangzhou Wahaha Group Co., Ltd, under Ethics Code Permit number WHH2022080401. Male Sprague-Dawley (SD) rats, aged five weeks, were procured from Shanghai SLAC Laboratory Animal Co., Ltd, and kept under specific pathogen-free (SPF) conditions, with controlled environmental parameters of 20 ± 5°C, 55% ± 5% humidity, and a 12 h light/dark cycle. During the experimental period, the rats were provided *ad libitum* access to feed and water.

The composition of the normal diet (ND) and HFD was consistent with that described in a prior study (22), with minor modifications. Animals were maintained on an ND (MD12031, composed of 10% calories from fat, 20% from protein, and 70% from carbohydrates) or an HFD (MD12033, composed of 60% calories from fat, 20% from protein, and 20% from carbohydrates). The composition of the experimental diets is shown in Supplementary Table 2.

**FIGURE 1:**
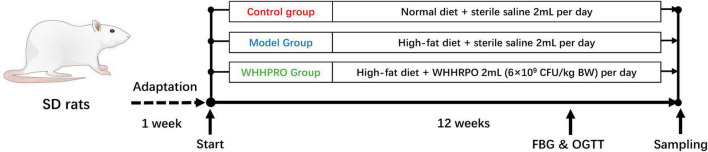
Schematic overview of the rat’s experimental design. After 1 week for adaptation, the rats were fed ND and HFD and were treated with saline or WHHPRO™ for 12 weeks. FBG and OGTT for all the rats were measured, after 11 weeks.

A schematic representation of the experimental design is provided in Figure 1. After 1 week of adaptation, a total of twenty-four male SD rats were randomly divided into three groups (*n* = 8 per group): control, model, and WHHPRO™ groups. The sample size of the present study was calculated based on a previous study (23). Rats in the control group were fed ND, while the rats in the model and WHHPRO™ groups were fed HFD. The rats in the control and model groups were orally administrated with 2 ml sterile saline and the rats in the WHHPRO™ group were orally administrated with 2 ml probiotic preparation dissolved in sterile saline (6 × 10^9^ CFU/kg body weight).

The body weight and food intake of the rats were recorded upon arrival and monitored weekly throughout the 12-week experimental period. Energy intakes were calculated based on caloric density values of 3.85 kcal/g and 5.24 kcal/g for the normal and high-fat diets, respectively. At the end of the study, the rats were fasted overnight and euthanized under ether anesthesia. Blood samples were collected from the heart and centrifuged at 3,500 × *g* at 4°C to obtain serum. The liver and epididymal fat were excised, weighed, and normalized to body weight (mg/g). Fresh feces, liver, ileal, and colonic samples were immediately stored at −80°C for further analysis.

### Oral glucose tolerance test (OGTT), fasting blood glucose (FBG), and insulin

At the 11-week time point, OGTT was performed according to the protocol of previous studies with some modifications (23). Prior to the OGTT, rats were fasted overnight for 10 h, from 8 pm to 10 am the following day. After fasting, rats were orally administered a glucose solution (2 g/kg), and blood samples were obtained from the tail vein at 0, 30, and 120 min after glucose loading. Prior to the administration of a glucose solution, FBG was tested. Blood glucose levels were determined promptly using an Accu-chek glucometer (Roche Diabetes Care GmbH, Mannheim, Germany). The fasting serum insulin concentration was assessed using a commercial assay kit according to the manufacturer’s instructions (product no. H203, Nanjing Jiancheng Bioengineering Institute, Nanjing, China). A homeostasis model assessment for insulin resistance (HOMA-IR) was used to estimate insulin resistance as follows: HOMA-IR = fasting insulin × FBG/22.5 (24).

### Lipids analysis

The concentrations of TG, TC, high-density lipoprotein cholesterol (HDL-C), and low-density lipoprotein cholesterol (LDL-C) were measured using an LW C400 automatic analyzer (LandWind, Shenzhen, China), following the manufacturer’s guidelines. Briefly, the blood samples were collected from the heart and the samples from the liver and fecal content were also collected, allowed to rest for 10–15 min at room temperature, and separated or homogenized by centrifugation at 3,500 × *g* for 15 min at 4°C, prior to analysis.

### Determination of SCFAs

Short-chain fatty acids (SCFAs) present in feces (freeze-dried) and serum samples were evaluated according to the previously described method with slight modifications (19). Briefly, around 200 mg of fecal samples were taken in 2 mL centrifuge tubes and soaked in 500 μL of saturated NaCl solution (serum samples were excluded from this step). The samples were then acidified with 4% (v/v) sulfuric acid solution (10%), followed by extraction with 800 μL diethyl ether. The supernatant was obtained by centrifugation at 14,000 × *g* for 10 min at 4°C. Dehydration of the supernatant was carried out by adding 250 mg of sodium sulfate, followed by filtration through a 0.22 μm filter. The analysis of SCFAs was conducted using the GC-MS platform (Agilent Technologies, Santa Clara, CA, USA), as previously described (19).

### Determination of BAs

Bile acids (BAs) present in feces (freeze-dried) and serum samples were evaluated according to the previously described method with slight modifications (25). Briefly, 50.0 mg fecal or liver samples were accurately weighed on ice, placed in a 2 mL Eppendorf tube, and soaked in 500 μL of 80% pre-cooled methanol/water mixture. The samples were then ground for 3 min, homogenized with a tissuelyzer (QIAGEN, TissueLyser II, Hilden, Germany) at 20 Hz for 2 min centrifugation at 10,000 × *g* for 15 min at 4°C, and the resulting supernatant was transferred to a UPLC-MS/MS (Agilent Technologies, Inc., Santa Clara, CA, USA) injection vial for analysis, as previously described (25). Total BAs were then calculated by adding all detected and measured BAs.

### Quantitative real-time PCR

Due to limited research funding, 6 samples from each group were randomly selected to analyze target gene expression. Total RNA was extracted from the tissue samples of animals using a Total RNA extraction reagent (HaoKe Biotechnology, Hangzhou, China). The extracted RNA was further reverse transcribed using All-in-One First-Strand Synthesis MasterMix (with dsDNase) (Yugong Biotechnology, Jiangsu, China). Real-time PCR was conducted in triplicate, following the manufacturer’s instructions for Taq SYBR^®^ Green q-PCR Premix (Yugong Biotechnology, Jiangsu, China). The primer sequences used for the PCR have been listed in Supplementary Table 3. *Gapdh* was selected as the housekeeping gene, and the relative expression of the target gene was quantified using the 2^–ΔΔCt^ method (26).

### 16S rRNA high-throughput sequencing

Due to limited research funding, 6 samples from each group were randomly selected to perform 16S rRNA high-throughput sequencing. DNA extraction was performed on fecal samples using the phenol/chloroform method. The V3-V4 regions of bacterial 16S rRNA amplicons were amplified using DNA primer sets and sequenced using the Illumina MiSeq platform, following the manufacturer’s standard instructions with 2 × 250-bp paired-end reads. After sequencing, the 16S rRNA reads were demultiplexed and quality-filtered using QIIME 2. The fecal microbiota was analyzed and characterized according to a previously published study (26). The sequencing data were deposited into the NCBI Sequence Read Archive (SRA) database under the accession number PRJNA988830.

### Statistical analysis

Statistical analysis was performed using the R software package (version 4.0.3). All data were conducted with multiple comparisons of the means using a one-way analysis of variance (ANOVA) followed by Tukey’s *post-hoc* test. To be considered statistically significant, differences had to have a *P*-value of less than 0.05.

## Results

### Effect of WHHPRO™ on body weight, food consumption, epididymal fat index, and liver index in HFD rats

**FIGURE 2:**
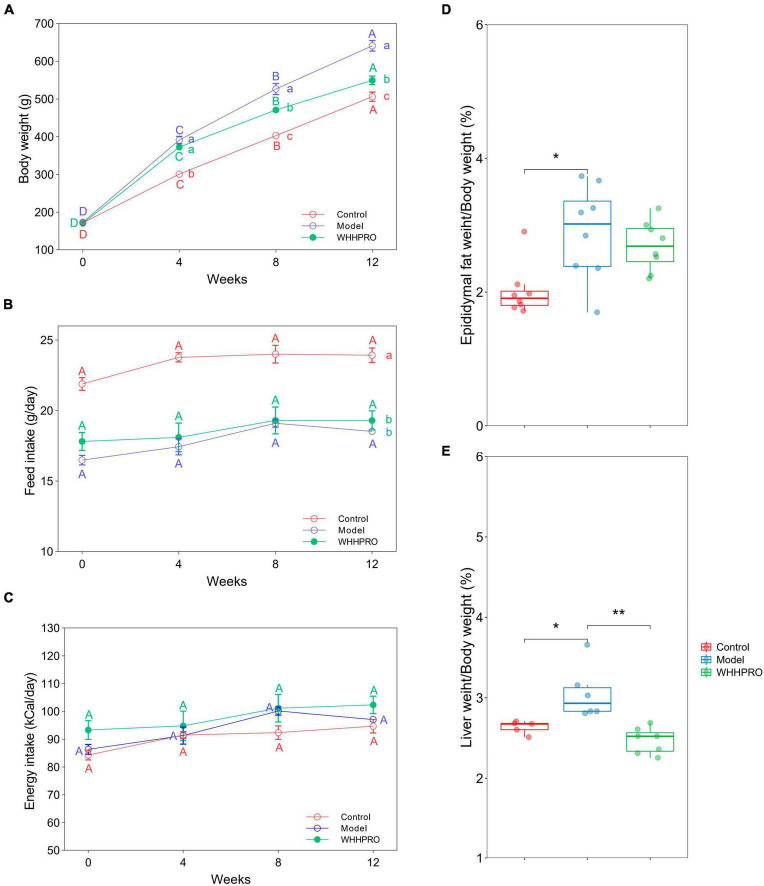
Effects of WHHPRO™ on body weight, food consumption, epididymal fat index, and liver index in HFD rats. **(A)** Body weight change. **(B)** Feed intake. **(C)** Energy intake. **(D)** Epididymal fat index. **(E)** Liver index. Columns with different letters differ significantly (*P* < 0.05). For scatter plots, data are expressed as medians ± 95% CI. *P < 0.05 and **P < 0.01. One-way ANOVA with Tukey’s *post-hoc* test for all groups.

The initial mean body weights were similar among the three groups (Figure 2A, *P* > 0.05), demonstrating the successful randomization and homogeneity of the study subjects. Rats in the model group had significantly higher weights at all-time points during the experiment period compared to the control group (*P* < 0.05). Administration of WHHPRO™ resulted in a significant reduction in body weight (*P* < 0.05). During the 12 weeks of the diet regimens, the rats in the control group had a significantly higher food intake than the other groups (Figure 2B, *P* < 0.05). As shown (Figure 2C), there was no significant difference in energy intake among the three groups (*P* > 0.05). We also investigated the effect of WHHPRO™ on the epididymal fat index and liver index, here, the significant increases in the epididymal fat index and liver index were observed after HFD (Figures 2D, E, *P* < 0.05). However, the administration of WHHPRO™ did not significantly affect the epididymal fat index in HFD rats (*P* > 0.05). Interestingly, we observed a significant reduction in the liver index in rats treated with WHHPRO™ compared to the model group (*P* < 0.05). Collectively, our results suggest that WHHPRO™ administration may have a beneficial effect on body weight and liver index. This finding suggests that WHHPRO™ may have a beneficial effect on obesity and related metabolic diseases, potentially through its interactions with hepatic lipid metabolism.

### Effect of WHHPRO™ on FBG, OGTT, HOMA-IR, serum lipid levels, and fasting insulin level in HFD rats

**FIGURE 3:**
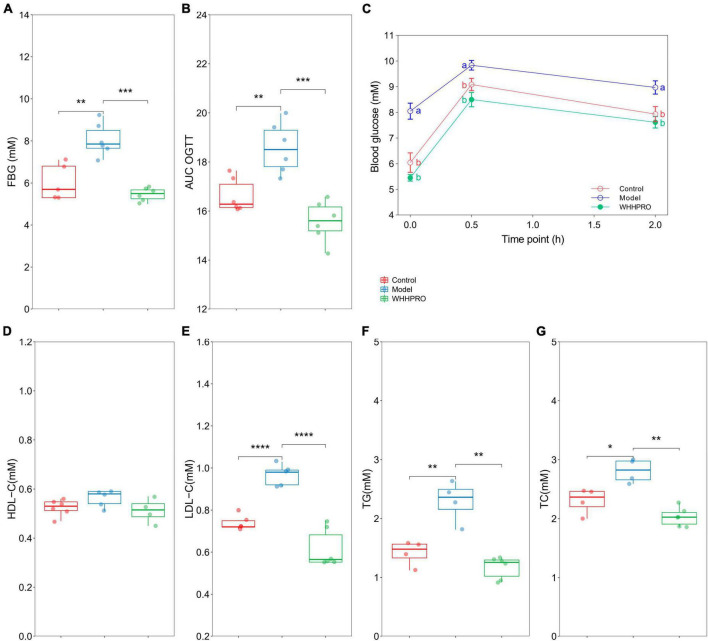
Effect of WHHPRO™ on FBG, OGTT, and serum lipid levels in HFD rats. **(A)** Fasting blood glucose (FBG) level. **(B)** The area under the curve (AUC) of the OGTT. **(C)** Blood glucose level before and after the oral glucose. **(D)** Serum high-density lipoprotein cholesterol (HDL-C) level. **(E)** Serum low-density lipoprotein cholesterol (LDL-C) level. **(F)** Serum triacylglycerol (TG) level. **(G)** Serum total cholesterol (TC) level. Columns with different letters differ significantly (*P* < 0.05). For scatter plots, data are expressed as medians ± 95% CI. *P < 0.05, **P < 0.01, ***P < 0.001 and ****P < 0.0001. One-way ANOVA with Tukey’s *post-hoc* test for all groups.

The FBG levels were significantly increased after 12 weeks of HFD (*P* < 0.01) and were reversed by WHHPRO™ administration (Figure 3A, *P* < 0.001). Rats in the model group had significantly higher blood glucose levels at all-time points during the OGTT compared to the control group (Figure 3C, *P* < 0.05), indicating impaired glucose tolerance. Consistent with the FBG data, administration of WHHPRO™ significantly reduced blood glucose levels during the OGTT (*P* < 0.05). Moreover, the area under the curve (AUC) of blood glucose levels during the OGTT was significantly increased in the model group compared to the control group (Figure 3B, *P* < 0.01), which was significantly reversed by treatment with WHHPRO™ (*P* < 0.001). Our data demonstrate that WHHPRO™ exerts the effects on blood glucose-lowering and improving glucose tolerance in HFD rats. Compared to the control group, rats in the model group had significantly higher fasting insulin levels and HOMA-IR which were reversed after WHHPRO™ intervention (Supplementary Figure 4, *P* < 0.0001).

In addition, the effects of WHHPRO™ on serum lipid levels in HFD rats were also evaluated (Figures 3D–G). The serum levels of LDL-C (*P* < 0.0001), TG (*P* < 0.01), and TC (*P* < 0.05) were significantly elevated after 12 weeks HFD, and which were significantly reduced after WHHPRO™ treatment (*P* < 0.01), suggesting a potential therapeutic application in the management of dyslipidemia. The effects of WHHPRO™ on the key lipid parameters in the liver and feces were also evaluated (Supplementary Table 4). As shown, Compared to the control group, rats in the model group had significantly higher levels of the key lipid parameters in the liver and feces (*P* < 0.0001). After the WHHPRO™ intervention, there was a significant increase in fecal lipid excretion (*P* < 0.0001) and a reduction in liver lipid accumulation (*P* < 0.0001). Collectively, our data support the potential therapeutic benefits of WHHPRO™ in the management of dyslipidemia-associated conditions.

### Effect of WHHPRO™ on SCFAs metabolism in HFD rats

**FIGURE 4:**
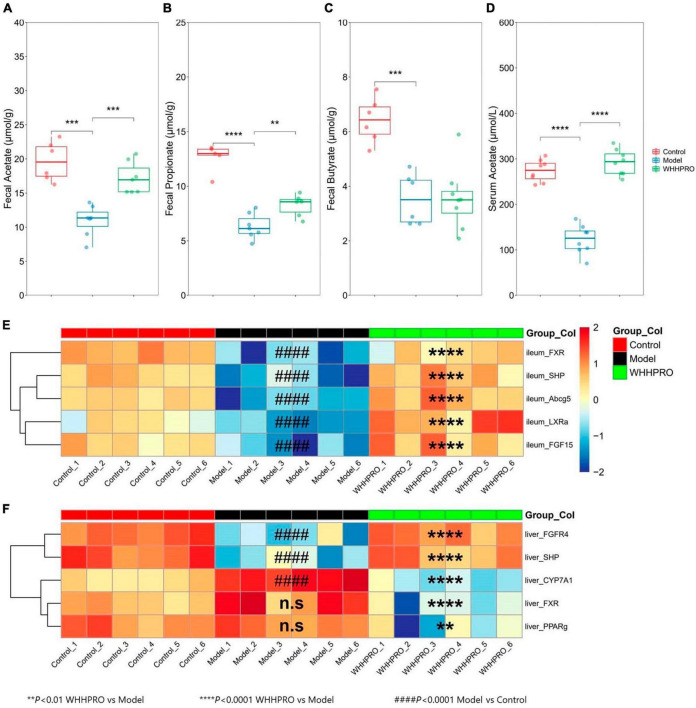
Effect of WHHPRO™ on SCFAs metabolism and the expression of *FXR* signaling target *gene* in HFD rats. **(A)** Fecal acetate level. **(B)** Fecal butyrate level. **(C)** Fecal propionate level. **(D)** Serum acetate level. *P < 0.05, **P < 0.01, ***P < 0.001 and ****P < 0.0001. Data are expressed as medians ± 95% CI. *FXR* signaling targets *gene* expression in the ileum **(E)** and liver **(F)**. ####*P* < 0.0001, model vs. control. **P < 0.01 and ****P < 0.0001, WHHPRO™ vs. model. One-way ANOVA with Tukey’s *post-hoc* test for all groups.

In this study, the concentrations of SCFAs in feces and serum were measured. As shown (Figures 4A–C), the levels of acetate (*P* < 0.001), propionate (*P* < 0.0001), and butyrate (*P* < 0.001) were significantly decreased after 12 weeks of HFD. Administration of WHHPRO™ resulted in a significant reversal of the HFD-induced decrease in acetate (*P* < 0.001) and propionate (*P* < 0.01) levels, suggesting a potential role of WHHPRO™ in modulating gut microbial fermentation and SCFA metabolism. Interestingly, we also observed a similar trend for acetate concentration in serum (Figure 4D, *P* < 0.0001). Our findings indicate that WHHPRO™ treatment may affect not only the SCFA production in the gut but also their systemic circulation and potential metabolic effects.

### Effect of WHHPRO™ on fecal and hepatic BAs in HFD rats

To explore the influence of WHHPRO™ on BA circulation, the changes in BA composition in the feces were analyzed (Table 1). Notably, the level of total fecal conjugated BAs in HFD rats was significantly increased and was normalized after WHHPRO™ treatment (*P* < 0.001) and compared with the model group, administration of WHHPRO™ significantly increased the total fecal unconjugated BAs (*P* < 0.001) and the total fecal BAs (*P* < 0.001). Further, the changes in the proportion of BA species were assessed. The levels of unconjugated primary BAs such as ω-MCA/*a*-MCA (*P* < 0.0001), b-MCA (*P* < 0.0001), HCA (*P* < 0.01), and CDCA (*P* < 0.01) were significantly increased after HFD (*P* < 0.01) and further increased after WHHPRO™ intervention, as well as unconjugated secondary BA DCA (*P* < 0.0001). Conversely, the levels of conjugated secondary BA GHDCA (*P* < 0.0001) and unconjugated secondary BA HDCA (*P* < 0.0001) were significantly decreased after HFD and further decreased after WHHPRO™ intervention (*P* < 0.0001). Moreover, the levels of conjugated primary BAs including T-b-MCA (*P* < 0.05) and TCA (*P* < 0.0001) were significantly increased after HFD and were reversed after WHHPRO™ intervention (*P* < 0.05), as well as 12-KDCA (*P* < 0.0001). Conversely, the unconjugated secondary BAs including ILCA (*P* < 0.0001) and LCA (*P* < 0.01) were found to be significantly decreased in the model group and were normalized after WHHPRO™ treatment (*P* < 0.01). These findings suggest that WHHPRO™ treatment may influence the metabolism and composition of BAs in the gut.

**TABLE 1:** Bile acid profiles in the feces of rats (μmol/mg).

Classification	BAs	Groups			*P*-values
		Control	Model	WHHPRO	
Primary BAs (unconjugated)	ω-MCA/*a*-MCA	77.22 ± 24.66^c^	331.13 ± 50.44^b^	1,113.56 ± 9.89^a^	< 0.00001
	b-MCA	31.17 ± 5.46^c^	449.22 ± 144.76^b^	1,081.74 ± 38.15^a^	< 0.00001
	HCA	0.51 ± 0.09^c^	6.79 ± 2.41^b^	12.69 ± 1.94^a^	0.003
	CA	14.39 ± 2.22^b^	711.28 ± 397.57^a^	529.86 ± 127.88^a^	0.023
	CDCA	0.87 ± 0.21^b^	15.78 ± 8.10^b^	76.42 ± 19.41^a^	0.004
	ApoCA	1.20 ± 0.11^b^	4.53 ± 1.04^a^	5.58 ± 0.81^a^	0.007
	3A-H-7-O-5B-CA	6.80 ± 2.47^b^	12.37 ± 1.70^b^	21.70 ± 3.74^a^	0.013
Primary BAs (conjugated)	T-b-MCA	1.60 ± 0.29^b^	10.14 ± 3.53^a^	1.36 ± 0.01^b^	0.022
	T-*a*-MCA	2.86 ± 0.47^ab^	1.56 ± 0.45^b^	4.40 ± 0.64^a^	0.013
	TCA	21.28 ± 1.48^b^	45.19 ± 4.14^a^	18.19 ± 2.26^b^	< 0.00001
	GCA	3.03 ± 1.02	1.81 ± 0.38	5.97 ± 2.74	0.258
	TCDCA	2.88 ± 0.59	1.85 ± 0.10	2.31 ± 0.29	0.220
Secondary BAs (unconjugated)	isoalloLCA	79.66 ± 12.87^a^	14.86 ± 1.14^b^	7.42 ± 0.73^b^	< 0.00001
	ILCA	45.81 ± 5.29^a^	12.02 ± 1.40^c^	23.65 ± 1.45^b^	< 0.00001
	LCA	282.53 ± 22.38^a^	87.23 ± 8.97^b^	278.15 ± 57.11^a^	0.006
	DCA	1,118.19 ± 226.47^c^	2,484.72 ± 462.50^b^	5,106.98 ± 286.76^a^	< 0.00001
	NorDCA	4.04 ± 1.09	4.76 ± 0.37	6.64 ± 1.38	0.239
	HDCA	2,488.54 ± 77.94^a^	958.99 ± 47.79^b^	496.69 ± 110.29^c^	< 0.00001
	UCA	15.90 ± 1.43	23.48 ± 6.22	110.99 ± 46.27	0.062
	MDCA	113.35 ± 1.17^a^	28.68 ± 2.71^b^	27.51 ± 1.18^b^	< 0.00001
	12-KDCA	390.43 ± 24.39^c^	890.94 ± 78.34^a^	661.80 ± 18.48^b^	< 0.00001
Secondary BAs (conjugated)	TUDCA	0.54 ± 0.05	1.16 ± 0.35	0.061 ± 0.02	0.018
	THDCA	2.19 ± 0.09^a^	2.59 ± 0.58^a^	0.63 ± 0.21^b^	0.009
	GHDCA	2.48 ± 0.06^a^	1.34 ± 0.25^b^	0.64 ± 0.20^c^	< 0.00001
	GDCA	1.17 ± 0.29^b^	2.64 ± 0.59^b^	6.10 ± 0.93^a^	0.001
Total conjugated BAs		38.03 ± 2.54^b^	68.26 ± 9.41^a^	40.18 ± 2.20^b^	0.008
Total unconjugated BAs		4,670.57 ± 317.93^b^	6,036.76 ± 1,189.05^b^	9,561.36 ± 499.98^a^	0.004
Total BAs		4,708.60 ± 320.47^b^	6,105.02 ± 1,179.64^b^	9,601.54 ± 502.18^a^	0.004

^a,b,c^One-way ANOVA or Kruskal Wallis tests were used to compare differences among the Control, Model, and WHHPRO groups.

In addition, the alterations in the BAs composition in liver samples were further analyzed (Table 2). The total hepatic BA pool size was significantly increased after 12 weeks of HFD (*P* < 0.0001) and was reversed after WHHPRO™ treatment (*P* < 0.0001). The level of total hepatic unconjugated BA was significantly decreased in HFD rats and was reversed after WHHPRO™ treatment (*P* < 0.001). Concomitantly, the levels of unconjugated primary BAs such as CA (*P* < 0.0001), ω-MCA/*a*-MCA (*P* < 0.01), and conjugated primary BA GCA (*P* < 0.05) were significantly decreased after HFD and reversed after WHHPRO™ treatment (*P* < 0.05). Conversely, the levels of conjugated primary BAs including TCA (*P* < 0.0001) and T-b-MCA (*P* < 0.01) were significantly increased after HFD and attenuated after WHHPRO™ treatment (*P* < 0.01). These findings indicated that WHHPRO™ treatment may have a beneficial impact on both gut and hepatic BA metabolism, offering potential therapeutic benefits for metabolic dysfunction associated with HFD feeding.

**TABLE 2:** Bile acid profiles in the liver of rats (μmol/mg).

Classification	BAs	Groups			*P*-values
		Control	Model	WHHPRO	
Primary BAs (unconjugated)	CA	0.48 ± 0.01^a^	0.14 ± 0.01^b^	0.46 ± 0.02^a^	< 0.00001
	b-MCA	0.31 ± 0.01	0.13 ± 0.01	0.23 ± 0.08	0.068
	ω-MCA/*a*-MCA	0.14 ± 0.01^a^	0.06 ± 0.01^b^	0.16 ± 0.02^a^	0.001
Primary BAs (conjugated)	TCA	57.98 ± 3.81^c^	136.13 ± 0.78^a^	87.65 ± 11.64^b^	< 0.00001
	T-*a*-MCA	7.55 ± 1.30	8.29 ± 0.62	10.49 ± 4.50	0.738
	T-b-MCA	4.83 ± 0.15^b^	13.76 ± 2.94^a^	5.64 ± 0.21^b^	0.009
	GCA	19.57 ± 6.06^a^	2.58 ± 0.19^b^	26.72 ± 12.97^a^	0.023
	TCDCA	3.63 ± 0.43	4.37 ± 0.05	6.36 ± 2.74	0.49
	GCDCA	0.81 ± 0.01	0.13 ± 0.01	2.34 ± 1.31	0.161
Secondary BAs (unconjugated)	HDCA	0.35 ± 0.07^a^	0.15 ± 0.01^b^	0.25 ± 0.02^ab^	0.048
	DCA	0.10 ± 0.02	0.10 ± 0.03	0.08 ± 0.02	0.803
Secondary BAs (conjugated)	THDCA	6.97 ± 0.42^a^	5.69 ± 0.12^a^	2.96 ± 0.70^b^	0.001
	GHDCA	3.06 ± 0.81	0.14 ± 0.02	1.86 ± 1.00	0.061
	TUDCA	0.59 ± 0.04	1.44 ± 0.10	1.33 ± 0.59	0.218
	GDCA	1.97 ± 0.61	0.18 ± 0.07	2.98 ± 1.62	0.192
	TLCA	0.52 ± 0.08	0.18 ± 0.05	0.50 ± 0.23	0.219
	GUDCA	0.13 ± 0.02	0.03 ± 0.01	0.35 ± 0.19	0.166
Total conjugated BAs (μmol/mg)		107.61 ± 10.17	172.92 ± 4.17	149.14 ± 37.71	0.175
Total unconjugated BAs (μmol/mg)		1.38 ± 0.09^a^	0.54 ± 0.06^b^	1.12 ± 0.12^a^	< 0.00001
Total BAs (μmol/mg)		108.99 ± 10.07	173.46 ± 4.11	150.26 ± 37.81	0.186
Total hepatic BA pool (mmol)^d^		1,783.26 ± 159.58^b^	4,249.77 ± 92.87^a^	2,133.69 ± 204.19^b^	< 0.00001

^a,b, c^One-way ANOVA or Kruskal Wallis tests were used to compare differences among the Control, Model, and WHHPRO groups.

^d^Total hepatic BA pool (mmol) = total BAs (μmol/mg) × liver weight (mg).

### Effect of WHHPRO™ on the expressions of FXR signaling target gene in HFD rats

Given the changes in the composition of BA species with known FXR agonist activity (LCA) and FXR antagonist activity (T-b-MCA), we further analyzed the subsequent effects on the FXR-FGF15 signaling pathway and the gene expressions of *Fxr* and its target genes in the ileum and liver were evaluated. As shown, the expressions of ileal *Fxr* and FXR-target gene, including *Shp* and *Fgf15*, were significantly decreased in the HFD rats (Figure 4D, *P* < 0.0001), and were restored by the administration of WHHPRO™ (*P* < 0.0001). The changes in the gene expressions of *Lxra* and *Abcg5* exhibited similar trends (Figure 4E, *P* < 0.0001). In contrast, the gene expressions of hepatic *Shp* and *Fgfr4* were significantly decreased in the HFD rats (Figure 4F, *P* < 0.0001) and were normalized after WHHPRO™ treatment (*P* < 0.0001). Additionally, the gene expression of *Cyp7a1*, a critical enzyme involved in BA synthesis, was significantly increased after 12 weeks of HFD (Figure 4F, *P* < 0.001) and was attenuated after WHHPRO™ treatment (*P* < 0.0001). Moreover, the gene expressions of hepatic *Fxr* and *Pparg* were significantly decreased after the administration of WHHPRO™ (Figure 4F, *P* < 0.01). Compared to the control group, the ileal gene expressions and liver *FGFR4* and *SHP* were not significantly changed in the WHHPRO™ group (Figure 4E, *P* > 0.05). Interestingly, the gene expressions of *CYP7A1*, *PPAR*γ, and *FXR* in the liver were significantly reduced after administration of WHHPRO™ (Figure 4F, *P* < 0.01). Collectively, our findings indicated that WHHPRO™ may alter the composition of the BA profiles and modulate the *FXR-FGF15* signaling.

### Effect of WHHPRO™ on the gut microbiome composition in HFD rats

**FIGURE 5:**
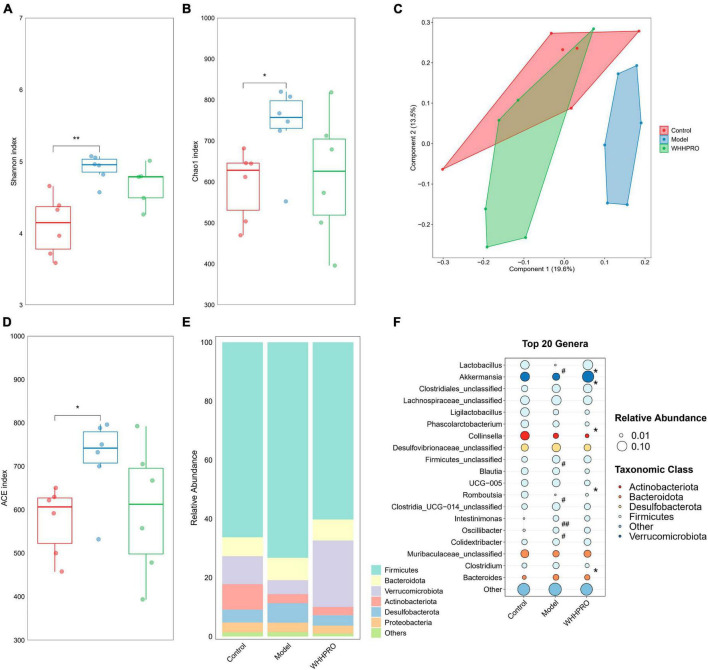
Effect of WHHPRO™ on the gut microbiome composition in HFD rats. Alpha diversity including the Shannon index **(A)**, Chao1 index **(B)**, ACE index **(D)**. Data are expressed as medians ± 95% CI. *P < 0.05, **P < 0.01. **(C)** Beta diversity is indicated by the principal coordination analysis (PCoA). **(E)** The relative abundance of gut microbial taxa at the phylum level. **(F)** The relative abundance of key differential genera. #*P* < 0.05 and ##*P* < 0.01 model vs. control. *P < 0.05, WHHPRO™ vs. model. One-way ANOVA with Tukey’s *post-hoc* test for all groups.

Consistent with the changes in the proportion of fecal BAs and SCFAs in HFD rats, the composition of the gut microbiota also changed after WHHPRO™ treatment. In this study, we evaluated the alpha diversity of the fecal microbiota using the Shannon index (Figure 5A), Chao1 index (Figure 5B), and ACE index (Figure 5D). The results revealed a significant increase in alpha diversity in the model group, compared to the control group (*P* < 0.05). To analyze the differences in gut microbiome composition, we performed principal coordinate analysis (PCoA) based on the Bray-Curtis distance. The data indicated a significant alteration in gut microbiome composition in HFD rats, with an evident separation between the control and model groups (Figure 5C, *P* < 0.01). Importantly, WHHPRO™ and control groups exhibited more overlapping regions, suggesting a similar compositional feature between the two groups. These findings revealed the modulatory effects of HFD on the gut microbiome composition and the potential of WHHPRO™ as an intervention to mitigate the impact of HFD on the gut microbiome composition.

At the phylum level, the abundance of Actinobacteriota was significantly decreased in the HFD rats (Figure 5E, *P* < 0.05), and the abundance of Verrucomicrobiota was significantly increased after WHHPRO™ treatment (*P* < 0.01). At the genus level (Figure 5F), the abundance of the *Lactobacillus* genus was significantly decreased in HFD rats (*P* < 0.05) and was reversed after WHHPRO™ treatment (*P* < 0.01). Interestingly, the abundance of the *Akkermansia* genus was significantly increased after WHHPRO™ treatment (*P* < 0.05). Furthermore, rats in the model group showed higher abundances of *unclassified Firmicutes*, *Intestinimonas*, and *Oscillibacter* genera (*P* < 0.05), while a lower abundance of *Romboutsia* genus (*P* < 0.05). Compared with the model group, the rats in the WHHPRO™ group showed lower abundances of *Phascolarctobacterium*, *UCG-005*, *Family-XIII-AD3011*, and *Clostridium* genera (*P* < 0.05). These findings provided further evidence of the potential of WHHPRO™ as a dietary intervention to modulate gut microbiome composition, especially in the case of increasing the abundance of *Lactobacillus* and *Akkermansia* genera, which were two dominant genera known for their beneficial effects on intestinal microbial ecology.

### Correlations analysis among bacteria, MS-related phenotypes, and metabolic parameters along the GLA

**FIGURE 6:**
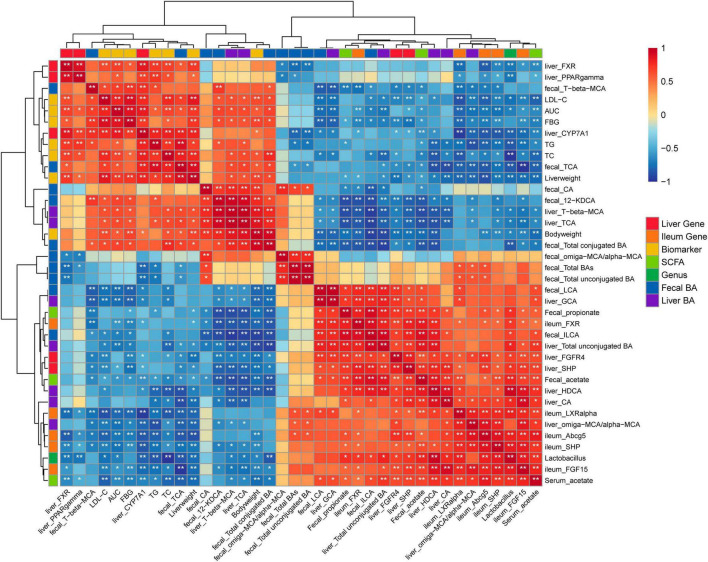
Heatmap of Spearman’s rank correlation analysis among bacterial genera, SCFAs, MS-related gene expressions, BAs, and key MS-related parameters. Positive correlations are shown using red color. Negative correlations are shown using blue color. *P < 0.05, **P < 0.01.

The significant correlations among bacterial genera, SCFAs, MS-related gene expressions, BAs, and key MS-related parameters were revealed using Spearman’s rank correlation analysis (Figure 6). Notably, the differences in fecal and hepatic BAs compositions, the fecal and serum SCFAs levels, the expression of FXR signaling target gene and the key MS-related biomarkers were associated with the alteration in the abundance of *Lactobacillus* genus (*P* < 0.05). The gene expressions of ileal *Fxr*, *Shp*, *Fgf15*, and hepatic *Fgfr4*, *Shp* were negatively correlated with the fecal 12-KDCA (*P* < 0.05). The gene expressions of ileal *Fxr*, *Fgf15*, *Shp*, *Liver X receptor a* (*Lxra*), *Abcg5*, and hepatic *Fgfr4*, *Shp* were negatively correlated with the fecal T-b-MCA (*P* < 0.05). The gene expression of ileal *Fxr* and hepatic *Fgfr4*, *Shp* were positively correlated with the fecal LCA and ILCA levels (*P* < 0.05). The key MS-related parameters, such as body weight, the levels of FBG, LDL-C, and TC, and the AUC were negatively correlated with the fecal LCA and ILCA levels (*P* < 0.05). In addition, the gene expression of hepatic *Cyp7a1* and the level of TG were negatively correlated with the fecal total unconjugated BAs (*P* < 0.05). The key MS-related parameters, such as the weights of the liver and body, the levels of TC, LDL-C, FBG, and the AUC were positively correlated with the fecal total conjugated BAs (*P* < 0.05).

## Discussion

A complex network among gut microbiota, BA metabolism, and MS-related parameters has revealed novel prophylactic interventions employing probiotics for metabolic diseases. Previous studies have reported the beneficial effects of a specific probiotic belonging to the *Lactobacillus* and *Bifidobacterium* genera on improving MS symptoms (20, 21, 27). Our study provided evidence supporting the beneficial effects of WHHPRO™, a multi-strain probiotic mixture, on improving MS symptoms, such as glucose tolerance, blood lipids, body weight, and liver index. In the current study, WHHPRO™ treatment also enriched the gut abundances of *Lactobacillus* and *Akkermansia*. In addition, the composition of BAs was altered and the accumulation of total hepatic BA pool size was reduced following WHHPRO™ treatment.

Obesity is often associated with MS. It is important to focus on weight control as a key aspect for the prevention and management of MS (28, 29). In line with previous studies (19, 30), our results found that HFD rats exhibited obvious symptoms of obesity, including higher body weights, increased epididymal fat index, and increased liver index. The administration of WHHPRO™ resulted in a reduction in body weight and liver index. Previous studies have reported that the administration of *Lactobacillus fermentum* CRL1446 and LGG can attenuate the increased body weight and liver index induced by HFD in mice (21, 31). Accordingly, our findings indicate that the supplementation of WHHPRO™ may have beneficial effects on obesity, especially in weight control.

According to the International Diabetes Federation (IDF), high levels of blood glucose and lipid abnormalities are important diagnostic criteria for MS (3). The OGTT has been widely used to assess glucose metabolism and insulin resistance (32). Consistent with previous studies (20, 33), in our study, the decreased FBG level and AUC value during the OGTT were observed after WHHPRO™ treatment in HFD rats, indicating that WHHPRO™ exerted a beneficial impact on glucose metabolism and insulin resistance. In addition, the increased levels of serum lipids, including LDL-C, TG, and TC, were reversed after WHHPRO™ treatment in HFD rats. Consistent with our findings, previous studies have reported that administration of *Lactobacillus fermentum* CRL1446 (21) and *Lactiplantibacillus pentosus* GSSK2 (34) can modify the serum lipid profile (decreased LDL-C, TG, and TC levels) in HFD rodents. Our findings suggest that the supplementation of WHHPRO™ may have the potential to modulate serum lipid metabolism, serving as a promising intervention for HFD-induced dyslipidemia.

It is known that BAs play an important role in host lipid metabolism. BAs are mainly synthesized in the liver and circulated in the intestine, where they facilitate the processes of fat digestion and absorption (17, 35). Therefore, reducing hepatic BA overload is a potential therapeutic application in the management of MS-related diseases (10, 36, 37). It has been demonstrated that unconjugated BAs exhibit higher hydrophobicity compared to conjugated BAs and are more easily excreted into feces (38). Probiotics, such as *Lactobacillus rhamnosus* GG can reduce the size of the hepatic BA pool by facilitating the conversion of conjugated BAs to unconjugated BAs and enhancing BA excretion in mice (36). Consistently, in the current study, the total fecal BA levels were increased and the total hepatic BA pool was decreased in HFD rats after WHHPRO™ treatment. Especially, the proportion of unconjugated BAs was increased both in the liver and feces. Therefore, our findings indicate that the supplementation with WHHPRO™ may result in an elevation in the proportion of unconjugated BAs, an enhancement of the excretion of BAs in feces, and a reduction in the hepatic BA overload.

Furthermore, the alterations in BA profiles contributed to lipid metabolism by regulating the activation of the FXR-FGF15 signaling pathway (17, 39–41). In the current study, after WHHPRO™ treatment, the BA composition was altered both in the feces and liver in HFD rats. The level of T-b-MCA, an FXR antagonist (15, 36), was decreased both in the liver and feces. Conversely, the levels of LCA and ILCA, FXR agonists (15), were increased in feces. Consistent with the changes in the composition of BA profiles, the FXR-FGF15 signaling along the GLA was activated, as evidenced by the increased gene expressions of the ileal *Fxr*, *Fgf15*, *Shp*, *Lxr*, and *Abcg5*, and the hepatic *Fgfr4* and *Shp*, and the decreased gene hepatic expressions of *Cyp7a1*, *Pparg*, and *Fxr* following the WHHPRO™ administration in HFD rats. Previous studies have also demonstrated that activation of intestinal FXR-FGF15 signaling in mice could reduce the hepatic BA pool by repressing hepatic *Cyp7a1* expression (36, 42). Apart from T-b-MCA, 12-KDCA may also be a potential FXR antagonist. Although there are currently no reports linking 12-KDCA to FXR antagonism, our research findings indicate a significant negative correlation between the level of 12-KDCA and the gene expression of *Fxr*. Further investigations are necessary to validate.

Growing evidence has revealed the significant involvement of the gut microbiota in the development of MS (3, 7, 43). Adverse changes in the diversity of intestinal bacteria are associated with metabolic disorders (33, 44). Our results supported this observation given that the HFD induced changes in gut microbiome composition, as reflected by an increased alpha diversity. Numerous studies have shown that the administration of some probiotic strains, such as *Lactobacillus* (33, 45) and *Bifidobacterium* (20, 46), can reshape the gut microbiota disorders induced by HFD. Consistently, in the current study, the gut microbial beta diversity of WHHPRO™ and control groups exhibited evident separations with the model group, highlighting the important role of WHHPRO™ consisting of four strains within *Lactobacillus* and *Bifidobacterium* in reshaping the gut microbial changes in HFD. Interestingly, the enriched abundances of *Lactobacillus* and *Akkermansia* genera were found after WHHPRO™ treatment. Numerous studies have documented close correlations between *Lactobacillus* and *Akkermansia* genera with the phenotypes of MS symptoms (19, 47, 48). Our findings indicate that WHHPRO™ treatment may improve MS phenotypes partly by shaping these associated genera.

Short-chain fatty acids (SCFAs), such as acetate, propionate, and butyrate, are mainly produced from dietary fiber as a result of gut microbial fermentation. The changes in the levels of SCFAs are associated with alterations in the gut microbial community (8). Administration of multi-strain probiotics with *Bifidobacterium* and *Lactobacillus* has been reported to result in changes in intestinal microbiota and the production of SCFAs in mice (49). Consistently, the present study found that the administration of WHHPRO™ resulted in an elevation in the abundance of *Lactobacillus* in HFD rats, one of the major SCFAs-producing genera (50). Moreover, the high level of fecal SCFAs, especially acetate and propionate, was observed. Our findings suggest that WHHPRO™ can promote the abundance of SCFA-producing microbes to elevate SCFA levels.

Furthermore, SCFAs have been demonstrated to impact multiple biological processes in various organs/tissues in hosts (8). Notably, a previous study has shown that dietary SCFA supplementation can reverse metabolic abnormalities induced by HFD in mice. This reversal is achieved through the downregulation of the peroxisome proliferator-activated receptor *g* (*Pparg*) expression (51). Consistently, the present study observed an elevation in fecal SCFAs, concurrently with an increase in serum acetate level and a downregulation of hepatic *Pparg* gene expression following the WHHPRO™ treatment in HFD rats. Collectively, these findings further highlight the potential impact of WHHPRO™ on hepatic lipid metabolism through the modulation of SCFAs.

**FIGURE 7:**
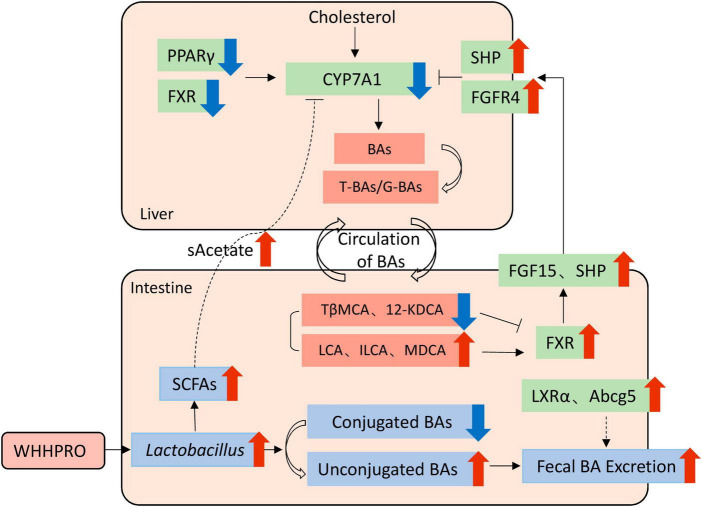
Schematic overview of the potential mechanisms by which WHHPRO™ supplementation can improve MS-related parameters through inhibiting hepatic BA synthesis and reducing the accumulation of total hepatic BA pool in HFD rats.

Therefore, based on our findings, we proposed a model to explain the underlying mechanisms (Figure 7). Specifically, WHHPRO™ treatment increased the abundance of *Lactobacillus* and *Akkermansia* genera in HFD rats. The reshaped gut microbiota played a profound effect on BA metabolism through different molecular mechanisms, which acted synergistically to alleviate symptoms associated with HFD-induced MS. First, the reshaped gut microbiota promoted the bio-transformation of conjugated BAs into unconjugated forms, consequently enhancing fecal BAs excretion. Second, the reshaped gut microbiota was observed to alter the composition of specific BAs, with decreased levels of FXR antagonists (T-b-MCA and 12-KDCA) and increased levels of FXR agonists (LCA and ILCA). These specific BAs further activated the FXR-FGF15 signaling pathway along the GLA and inhibited gene *Cyp7a1* expression and BA synthesis. Third, the increased abundance of the *Lactobacillus* genus promoted the production of SCFAs, which reached the liver via systemic circulation, thus inhibiting the gene expression of *Cyp7a1*. This proposed underlying mechanism will be investigated further.

The current study has several limitations. First, the intestinal barrier plays a pivotal role as the guardian of our health, shielding the liver from the influx of substantial quantities of bacterial compounds, and the dysfunction of the intestinal barrier is responsible for the pathogenesis of liver disease (52, 53). Probiotics are well acknowledged to improve intestinal barrier function, thus alleviating liver inflammation (54, 55). However, the intestinal barrier function and the association with WHHRPO™, gut microbiota, and liver function were not assessed, which should be investigated in the future. Second, the GLA is a complex bilateral network (11, 13), thereby we cannot simply conclude the pivotal roles of bile acid metabolism and FXR signaling in the alleviation of MS symptoms by WHHPRO™. The causal effects will be investigated further.

In conclusion, our current findings demonstrated that WHHPRO™ alleviated the key MS-related indicators in an HFD rat model, including improved glucose tolerance, blood lipid, body weight, and liver index. Importantly, these beneficial effects of WHHPRO™ were closely associated with the modulation of bile acid metabolism and the FXR-FGF15 signaling along the GLA, gut microbiome composition, and SCFA productions. The present study highlights the potential of WHHPRO™ supplementation as a promising therapeutic strategy for improving MS symptoms.

## Data availability statement

The original contributions presented in this study are publicly available. This data can be found here: https://www.ncbi.nlm.nih.gov, BioProject: PRJNA988830.

## Ethics statement

The animal studies were approved by the Animal Care and Use Committee of Hangzhou Wahaha Group Co., Ltd. The studies were conducted in accordance with the local legislation and institutional requirements. Written informed consent was obtained from the owners for the participation of their animals in this study.

## Author contributions

CC: Conceptualization, Formal analysis, Investigation, Writing – original draft, Writing – review & editing. KG: Conceptualization, Investigation, Methodology, Validation, Visualization, Writing – original draft, Writing – review & editing. ZC: Data curation, Investigation, Methodology, Validation, Writing – original draft. QZ: Investigation, Methodology, Writing – original draft. XK: Investigation, Methodology, Writing – original draft. BM: Supervision, Writing – original draft, Writing – review & editing. QF: Methodology, Resources, Writing – original draft. YL: Resources, Supervision, Writing – review & editing. SC: Funding acquisition, Resources, Supervision, Writing – review & editing.

## References

[1] Metascreen Writing Committee. The metabolic syndrome is a risk indicator of microvascular and macrovascular complications in diabetes: results from Metascreen, a multicenter diabetes clinic–based survey. *Diabetes Care.* (2006) 29:2701–7. 10.2337/dc06-0942 17130208

[2] Kapar FS Ciftci G. The effects of curcumin and *Lactobacillus* acidophilus on certain hormones and insulin resistance in rats with metabolic syndrome. *J Diabetes Metab Disord.* (2020) 19:907–14. 10.1007/s40200-020-00578-1 33553015 PMC7843847

[3] Tenorio-Jimenez C Martinez-Ramirez M Gil A Gomez-Llorente C. Effects of probiotics on metabolic syndrome: a systematic review of randomized clinical trials. *Nutrients.* (2020) 12:124–36. 31906372 10.3390/nu12010124 PMC7019472

[4] Qin J Li R Raes J Arumugam M Burgdorf KS Manichanh C A human gut microbial gene catalogue established by metagenomic sequencing. *Nature.* (2010) 464:59–65. 20203603 10.1038/nature08821 PMC3779803

[5] Backhed F Manchester JK Semenkovich CF Gordon JI. Mechanisms underlying the resistance to diet-induced obesity in germ-free mice. *Proc Natl Acad Sci USA.* (2007) 104:979–84. 17210919 10.1073/pnas.0605374104 PMC1764762

[6] Mazidi M Rezaie P Kengne AP Mobarhan MG Ferns GA. Gut microbiome and metabolic syndrome. *Diabetes Metab Syndr.* (2016) 10(2 Suppl. 1):150–7. 10.1016/j.dsx.2016.01.024 26916014

[7] Wang J Zhu N Su X Gao Y Yang R. Gut-microbiota-derived metabolites maintain gut and systemic immune homeostasis. *Cells.* (2023) 12:793–819. 36899929 10.3390/cells12050793 PMC10000530

[8] Koh A De Vadder F Kovatcheva-Datchary P Backhed F. From dietary fiber to host physiology: short-chain fatty acids as key bacterial metabolites. *Cell.* (2016) 165:1332–45. 27259147 10.1016/j.cell.2016.05.041

[9] Salminen SJ Gueimonde M Isolauri E. Probiotics that modify disease risk. *J Nutr.* (2005) 135:1294–8. 15867327 10.1093/jn/135.5.1294

[10] Abdi M Esmaeili Gouvarchin Ghaleh H Ranjbar R. *Lactobacilli* and *Bifidobacterium* as anti-atherosclerotic agents. *Iran J Basic Med Sci.* (2022) 25:934–46. 10.22038/IJBMS.2022.63860.14073 36159325 PMC9464336

[11] Liu HX Keane R Sheng L Wan YJ. Implications of microbiota and bile acid in liver injury and regeneration. *J Hepatol.* (2015) 63:1502–10. 10.1016/j.jhep.2015.08.001 26256437 PMC4654653

[12] Chiang JY Ferrell JM. Up to date on cholesterol 7 alpha-hydroxylase (CYP7A1) in bile acid synthesis. *Liver Res.* (2020) 4:47–63. 10.1016/j.livres.2020.05.001 34290896 PMC8291349

[13] Pushpass RG Alzoufairi S Jackson KG Lovegrove JA. Circulating bile acids as a link between the gut microbiota and cardiovascular health: impact of prebiotics, probiotics and polyphenol-rich foods. *Nutr Res Rev.* (2022) 35:161–80. 10.1017/S0954422421000081 33926590

[14] Russell DW. The enzymes, regulation, and genetics of bile acid synthesis. *Annu Rev Biochem.* (2003) 72:137–74. 12543708 10.1146/annurev.biochem.72.121801.161712

[15] Xiao Y Zhou K Lu Y Yan W Cai W Wang Y. Administration of antibiotics contributes to cholestasis in pediatric patients with intestinal failure via the alteration of FXR signaling. *Exp Mol Med.* (2018) 50:1–14. 10.1038/s12276-018-0181-3 PMC6269533 30504803

[16] Gonzalez FJ Jiang C Bisson WH Patterson AD. Inhibition of farnesoid X receptor signaling shows beneficial effects in human obesity. *J Hepatol.* (2015) 62:1234–6. 25747705 10.1016/j.jhep.2015.02.043 PMC6346267

[17] Duan Y Zhang F Yuan W Wei Y Wei M Zhou Y Hepatic cholesterol accumulation ascribed to the activation of ileum Fxr-Fgf15 pathway inhibiting hepatic Cyp7a1 in high-fat diet-induced obesity rats. *Life Sci.* (2019) 232:116638–116666. 10.1016/j.lfs.2019.116638 31288013

[18] Iqbal UH Westfall S Prakash S. Novel microencapsulated probiotic blend for use in metabolic syndrome: design and in-vivo analysis. *Artif Cells Nanomed Biotechnol.* (2018) 46(Suppl. 3):S116–24. 10.1080/21691401.2018.1489270 30033770

[19] Zhong H Abdullah, Deng L Zhao M Tang J Liu T Probiotic-fermented blueberry juice prevents obesity and hyperglycemia in high fat diet-fed mice in association with modulating the gut microbiota. *Food Funct.* (2020) 11:9192–207. 10.1039/d0fo00334d 33030465

[20] Le Barz M Daniel N Varin TV Naimi S Demers-Mathieu V Pilon G In vivo screening of multiple bacterial strains identifies *Lactobacillus rhamnosus* Lb102 and *Bifidobacterium animalis ssp. lactis* Bf141 as probiotics that improve metabolic disorders in a mouse model of obesity. *FASEB J.* (2019) 33:4921–35. 10.1096/fj.201801672R 30596521

[21] Russo M Marquez A Herrera H Abeijon-Mukdsi C Saavedra L Hebert E Oral administration of *Lactobacillus fermentum* CRL1446 improves biomarkers of metabolic syndrome in mice fed a high-fat diet supplemented with wheat bran. *Food Funct.* (2020) 11:3879–94. 10.1039/d0fo00730g 32421119

[22] Gallou-Kabani C Vige A Gross MS Rabes J Boileau C Larue-Achagiotis C C57BL/6J and A/J mice fed a high-fat diet delineate components of metabolic syndrome. *Obesity.* (2007) 15:1996–2005. 10.1038/oby.2007.238 17712117

[23] Senaphan K Kukongviriyapan U Sangartit W Pakdeechote P Pannangpetch P Prachaney P Ferulic acid alleviates changes in a rat model of metabolic syndrome induced by high-carbohydrate, high-fat diet. *Nutrients.* (2015) 7:6446–64. 10.3390/nu7085283 26247970 PMC4555122

[24] Haffner SM Greenberg AS Weston WM Chen H Williams K Freed MI. Effect of rosiglitazone treatment on nontraditional markers of cardiovascular disease in patients with type 2 diabetes mellitus. *Circulation.* (2002) 106:679–84. 12163427 10.1161/01.cir.0000025403.20953.23

[25] Want EJ Coen M Masson P Keun HC Pearce JT Reily MD Ultra performance liquid chromatography-mass spectrometry profiling of bile acid metabolites in biofluids: application to experimental toxicology studies. *Anal Chem.* (2010) 82:5282–99. 10.1021/ac1007078 20469835

[26] Gao K Farzi A Ke X Yu Y Chen C Chen S Oral administration of *Lactococcus lactis* WHH2078 alleviates depressive and anxiety symptoms in mice with induced chronic stress. *Food Funct.* (2022) 13:957–69. 10.1039/d1fo03723d 35006225

[27] Park S Kang J Choi S Park H Hwang E Kang Y Cholesterol-lowering effect of *Lactobacillus rhamnosus* BFE5264 and its influence on the gut microbiome and propionate level in a murine model. *PLoS One.* (2018) 13:e0203150. 10.1371/journal.pone.0203150 30153290 PMC6112659

[28] Gobato AO Vasques AC Zambon MP Barros Filho Ade A Hessel G. Metabolic syndrome and insulin resistance in obese adolescents. *Rev Paulista Pediatr.* (2014) 32:55–62. 10.1590/S0103-05822014000100010 PMC4182990 24676191

[29] Després JP Lemieux I. Abdominal obesity and metabolic syndrome. *Nature.* (2006) 444:881–7. 17167477 10.1038/nature05488

[30] Lin H An Y Tang H Wang Y. Alterations of bile acids and gut microbiota in obesity induced by high fat diet in rat model. *J Agric Food Chem.* (2019) 67:3624–32. 30832480 10.1021/acs.jafc.9b00249

[31] Ritze Y Bárdos G Claus A Ehrmann V Bergheim I Schwiertz A Lactobacillus rhamnosus GG protects against non-alcoholic fatty liver disease in mice. *PLoS One.* (2014) 9:e80169. 10.1371/journal.pone.0080169 24475018 PMC3903470

[32] Stumvoll M Mitrakou A Pimenta W Jenssen T Yki-Järvinen H Van Haeften T Use of the oral glucose tolerance test to assess insulin release and insulin sensitivity. *Diabetes Care.* (2000) 23:295–301. 10868854 10.2337/diacare.23.3.295

[33] Zheng F Wang Z Stanton C Ross RP Zhao J Zhang H Lactobacillus rhamnosus FJSYC4-1 and Lactobacillus reuteri FGSZY33L6 alleviate metabolic syndrome via gut microbiota regulation. *Food Funct.* (2021) 12:3919–30. 10.1039/d0fo02879g 33977963

[34] Khanna S Bishnoi M Kondepudi KK Shukla G. Synbiotic (Lactiplantibacillus pentosus GSSK2 and isomalto-oligosaccharides) supplementation modulates pathophysiology and gut dysbiosis in experimental metabolic syndrome. *Sci Rep.* (2021) 11:21397–413. 10.1038/s41598-021-00601-2 34725349 PMC8560755

[35] Ridlon JM Kang DJ Hylemon PB. Bile salt biotransformations by human intestinal bacteria. *J Lipid Res.* (2006) 47:241–59. 16299351 10.1194/jlr.R500013-JLR200

[36] Liu Y Chen K Li F Gu Z Liu Q He L Probiotic *Lactobacillus rhamnosus* gg prevents liver fibrosis through inhibiting hepatic bile acid synthesis and enhancing bile acid excretion in mice. *Hepatology.* (2020) 71:2050–66. 10.1002/hep.30975 31571251 PMC7317518

[37] Trauner M Fuchs CD Halilbasic E Paumgartner G. New therapeutic concepts in bile acid transport and signaling for management of cholestasis. *Hepatology.* (2017) 65:1393–404. 10.1002/hep.28991 27997980

[38] Geng W Lin J. Bacterial bile salt hydrolase: an intestinal microbiome target for enhanced animal health. *Anim Health Res Rev.* (2016) 17:148–58. 10.1017/S1466252316000153 28155801

[39] Mudaliar S Henry RR Sanyal AJ Morrow L Marschall HU Kipnes M Efficacy and safety of the farnesoid X receptor agonist obeticholic acid in patients with type 2 diabetes and nonalcoholic fatty liver disease. *Gastroenterology.* (2013) 145:574–82. 23727264 10.1053/j.gastro.2013.05.042

[40] Inagaki T Choi M Moschetta A Peng L Cummins CL McDonald JG Fibroblast growth factor 15 functions as an enterohepatic signal to regulate bile acid homeostasis. *Cell Metab.* (2005) 2:217–25. 16213224 10.1016/j.cmet.2005.09.001

[41] Katafuchi T Makishima M. Molecular basis of bile acid-FXR-FGF15/19 signaling axis. *Int J Mol Sci.* (2022) 23:6046–66. 10.3390/ijms23116046 35682726 PMC9181207

[42] Modica S Petruzzelli M Bellafante E Murzilli S Salvatore L Celli N Selective activation of nuclear bile acid receptor FXR in the intestine protects mice against cholestasis. *Gastroenterology.* (2012) 142:355–65.e1–4. 10.1053/j.gastro.2011.10.028 22057115

[43] Lin K Zhu L Yang L. Gut and obesity/metabolic disease: focus on microbiota metabolites. *Med Comm.* (2022) 3:171–92. 10.1002/mco2.171 36092861 PMC9437302

[44] Creely SJ McTernan PG Kusminski CM Fisher f M Da Silva NF Khanolkar M Lipopolysaccharide activates an innate immune system response in human adipose tissue in obesity and type 2 diabetes. *Am J Physiol Endocrinol Metab.* (2007) 292:E740–7. 17090751 10.1152/ajpendo.00302.2006

[45] Yang B Zheng F Stanton C Ross RP Zhao J Zhang H Lactobacillus reuteri FYNLJ109L1 attenuating metabolic syndrome in mice via gut microbiota modulation and alleviating inflammation. *Foods.* (2021) 10:2081–94. 10.3390/foods10092081 34574191 PMC8469823

[46] Uusitupa HM Rasinkangas P Lehtinen MJ Mäkelä SM Airaksinen K Anglenius H Bifidobacterium animalis subsp. lactis 420 for metabolic health: review of the research. *Nutrients.* (2020) 12:892–909. 10.3390/nu12040892 32218248 PMC7230722

[47] Li J Lin S Vanhoutte PM Woo CW Xu A. *Akkermansia* muciniphila protects against atherosclerosis by preventing metabolic endotoxemia-induced inflammation in apoe-/- mice. *Circulation.* (2016) 133:2434–46. 10.1161/CIRCULATIONAHA.115.019645 27143680

[48] Zhang H Ma W Sun Z Zhu C Werid GM Ibrahim YM Abundance of *Lactobacillus* in porcine gut microbiota is closely related to immune response following PRRSV immunization. *Vet Microbiol.* (2021) 259:109134–109144. 10.1016/j.vetmic.2021.109134 34087673

[49] Zhang C Wang L Liu X Wang G Guo X Liu X The different ways multi-strain probiotics with different ratios of bifidobacterium and lactobacillus relieve constipation induced by loperamide in mice. *Nutrients.* (2023) 15:4230–51. 10.3390/nu15194230 37836514 PMC10574055

[50] LeBlanc JG Chain F Martin R Bermudez-Humaran LG Courau S Langella P. Beneficial effects on host energy metabolism of short-chain fatty acids and vitamins produced by commensal and probiotic bacteria. *Microb Cell Fact.* (2017) 16:79–89. 10.1186/s12934-017-0691-z 28482838 PMC5423028

[51] den Besten G Bleeker A Gerding A van Eunen K Havinga R van Dijk TH Short-chain fatty acids protect against high-fat diet-induced obesity via a PPARgamma-dependent switch from lipogenesis to fat oxidation. *Diabetes.* (2015) 64:2398–408. 10.2337/db14-1213 25695945

[52] Pabst O Hornef MW Schaap FG Cerovic V Clavel T Bruns T. Gut-liver axis: barriers and functional circuits. *Nat Rev Gastroenterol Hepatol.* (2023) 20:447–61. 37085614 10.1038/s41575-023-00771-6

[53] Chopyk DM Grakoui A. Contribution of the intestinal microbiome and gut barrier to hepatic disorders. *Gastroenterology.* (2020) 159:849–63. 32569766 10.1053/j.gastro.2020.04.077 PMC7502510

[54] WuYZhangXLiuXLiYHanDPiY Strain specificity of lactobacilli with promoted colonization by galactooligosaccharides administration in protecting intestinal barriers during *Salmonella* infection. *J Adv Res.* (2023). 10.1016/j.jare.2023.03.001 [Epub ahead of print].36894120

[55] Martin-Gallausiaux C Garcia-Weber D Lashermes A Larraufie P Marinelli L Teixeira V Akkermansia muciniphila upregulates genes involved in maintaining the intestinal barrier function via ADP-heptose-dependent activation of the ALPK1/TIFA pathway. *Gut Microbes.* (2022) 14:2110639. 10.1080/19490976.2022.2110639 36036242 PMC9427033

