# Supplementary material for: The supplementation of the multi-strain probiotics WHHPRO™ alleviates high-fat diet-induced metabolic symptoms in rats via gut-liver axis
Source: Front Nutr. 2024 Jan 11;10:1324691. doi: 10.3389/fnut.2023.1324691 (PMC10808617; doi:10.3389/fnut.2023.1324691)
Supplement: Supplementary file 1 [file Data_Sheet_1.docx]

**Supplementary information**

**The supplementation of the multi-strain probiotics WHHPRO^TM^** **alleviates high-fat diet-induced metabolic symptoms in rats via gut-liver axis**

**Running title: WHHPRO^TM^ alleviates metabolic symptoms in HFD rats**

Cailing Chen^a, b, c^, Kan Gao^a, b, c,^ *, Zuoguo Chen^a, b, c^, Qiwen Zhang^a, b, c^, Xueqin Ke^a, b, c^, Bingyong Mao^d^, Qiuling Fan^a, b, c^, Yanjun Li^a, b, c^, Su Chen^a, b, c,^ *

^a^ *Key Laboratory of Food And Biological Engineering of Zhejiang Province, Hangzhou 310018, P.R. China*

^b^ *Research and Development Department, Hangzhou Wahaha Group Co., Ltd, Hangzhou 310018, P.R. China*

^c^ *Hangzhou Wahaha Technology Co., Ltd, Hangzhou 310018, P.R. China*

*^d^ School of Food Science and Technology, Jiangnan University, Wuxi 214122, China*

**First Author:**

Cailing Chen, Ph.D.

Email Address: chencailing@wahaha.com.cn

***Co-corresponding Author:**

Kan Gao, Ph.D., Associate Professor

Email Address: kevingogh911@hotmail.com

Su Chen, Ph.D., Associate Professor

Email Address: chensu@wahaha.com.cn

**Supplementary methods**

**Zebrafish experiment design**

Zebrafish husbandry and care of embryos and larvae were performed according to the previous study [1-2] with some modifications. Briefly, embryos were maintained in standard conditions at 28 °C (200 mg/L instant ocean salt in deionized water; pH 6.5-8.5; conductivity 450-550 μS/cm and hardness 50-100mg/L CaCO_3_). Zebraﬁsh larvae were provided by Hunter Biotechnology Co., Ltd, and the housing facilities of these zebrafish were accredited by the Association for Assessment and Accreditation of Laboratory Animal Care (AAALAC, accession number 001458). The experimental sample size and the age of the animals were determined according to the previous study [3].

The timeline of the experiments was determined according to the previous study [4] with some modifications. Wild-type zebrafish were used to evaluate the blood glucose-lowing (BG-L) and total cholesterol-lowering (TC-L) effects and the albino zebrafish were used to evaluate the triglycerides-lowering (TG-L) effect. The 5 days post-fertilization (dpf) larvae were randomly divided into six groups as follows (n=30 each): control, model, WHH2644, WHH2276, WHH3906, and WHH1155. The control group was fed a normal diet, and treated with 1% DMSO as vehicle control. Others were fed a high-fat diet with 1.5 mg/ml egg yolk (HFD-Y) provided by Zhejiang Aige Biotechnology Co., Ltd (Lot N: 20200809, China) and a high-carbohydrate diet (30 mg/ml glucose, HCD-G). Treatment groups received dietary supplementation of probiotics at a concentration of 10^8^ CFU/ml according to previous studies [5-6]. After being incubated for 48 h, larvae were collected and observed, and the general status of each group was recorded.

**Evaluations of zebrafish cholesterol levels**

Wild-type zebrafish were incubated in breeding water containing 0.1 ppm CholEsteryl BODIPY™ 542/563 C11 (Invitrogen, Lot N: 2291600, USA), at the 32nd hour. After 48 hours, ﬁsh were lethally anesthetized, and ten zebrafish were randomly sampled from each group, photographed using an electric focusing continuous amplification fluorescence microscope (AZ100, Nikon, Japan) and the fluorescence intensity was calculated using the NIS‑Elements BR 3.2 software (Nikon Corporation, Tokyo, Japan).

**Evaluations of zebrafish triglyceride levels**

Wild-type zebrafish were stained with Oil Red O (Lot N: SHBN4926, Sigma, USA) using the method described by the previous study [7]. Ten zebrafish were randomly sampled from each group, and photographed using a dissecting microscope (SZX7, OLYMPUS, Japan), and intravascular triglyceride concentration was analyzed using the Image-Pro Plus 6.0 (Media Cybernetics. Inc, Washington Street, USA).

**Evaluations of zebrafish blood glucose levels**

Ten albino zebrafish were randomly sampled from each group and the blood glucose levels were measured immediately with an Accu-chek glucometer (Roche Diabetes Care GmbH, Mannheim, Germany), following the manufacturer’s instructions.

**References**

[1] Haldi M, Ton C, Seng W L, et al. Human melanoma cells transplanted into zebrafish proliferate, migrate, produce melanin, form masses and stimulate angiogenesis in zebrafish[J]. Angiogenesis, 2006, 9: 139-151.

[2] Zhou, Juan, et al. "Rapid analysis of hypolipidemic drugs in a live zebrafish assay." Journal of Pharmacological and Toxicological Methods 72 (2015): 47-52.

[3] Quinlivan, Vanessa H., and Steven A. Farber. "Lipid uptake, metabolism, and transport in the larval zebrafish." Frontiers in endocrinology 8 (2017): 319.

[4] [1]Yu-Shi LiuMing-Hao YuanCun-Yan ZhangHong-Mei LiuJuan-Ru LiuAi-Ling WeiQiang YeBin ZengMei-Feng LiYi-Ping GuoLi Guo. "Puerariae Lobatae radix flavonoids and puerarin alleviate alcoholic liver injury in zebrafish by regulating alcohol and lipid metabolism." Biomedicine & pharmacotherapy =: Biomedecine & pharmacotherapie 134.1(2021).

[5] Hu, Chenyan, et al. "Probiotic intervention mitigates the metabolic disturbances of perfluorobutanesulfonate along the gut-liver axis of zebrafish." Chemosphere 284 (2021): 131374.

[6] Falcinelli, Silvia, et al. "Lactobacillus rhamnosus lowers zebrafish lipid content by changing gut microbiota and host transcription of genes involved in lipid metabolism." Scientific reports 5.1 (2015): 1-11.

[7] Liu, Yu-Shi, et al. "Puerariae Lobatae radix flavonoids and puerarin alleviate alcoholic liver injury in zebrafish by regulating alcohol and lipid metabolism." Biomedicine & Pharmacotherapy 134 (2021): 111121.

**Supplementary tables**

**Table S1** The detailed composition of WHHPRO^TM^

| **Strains** | **Proportions**  **(%)** | **Species** | **Origin** | **Collection**  **Location** | **BSH Activity(mm)^1^** |
| --- | --- | --- | --- | --- | --- |
| WHH2276 | 25 | *Bifidobacterium longum* | Infant feces | Hangzhou | 32.75±0.75 |
| WHH3906 | 25 | *Limosilactobacillus fermentum* | Butter | Tibet | 11.50±0.00 |
| WHH2644 | 25 | *Limosilactobacillus fermentum* | Yogurt | Xinjiang | 11.50±0.00 |
| WHH1155 | 25 | *Lacticaseibacillus rhamnosus* | Cheese | Inner Mongolia | 11.75±0.25 |

^1^ The data are presented as means ± SEM.

**Table S2.** The compositions of the experimental diets (g/100g)

| **Diet Composition** | **ND** | **HFD** |
| --- | --- | --- |
| Energy(kcal) | 385 | 524 |
| Carbohydrates (E %) | 70 | 20 |
| Protein (E %) | 20 | 20 |
| Fat (E %) | 10 | 60 |
| Casein (g) | 18.96 | 25.85 |
| Soybean oil (g) | 2.37 | 3.23 |
| Lard oil (g) | 1.90 | 31.66 |
| Corn starch (g) | 29.86 | 0 |
| Maltodextrin (g) | 3.32 | 16.15 |
| Sucrose (g) | 33.18 | 8.89 |
| Cellulose (g) | 4.72 | 6.46 |
| Minerals (g) | 4.27 | 5.82 |
| Multi-vitamins (g) | 0.95 | 1.29 |
| L-cysteine (g) | 0.28 | 0.39 |
| Choline bitartrate (g) | 0.19 | 0.26 |

E%, energy percentage.

ND, normal diet, HFD, high-fat diet.

Caloric density values: 3.85 kcal/g for ND and 5.24 kcal/g for HFD.

**Table S3** Primer information

| Gene primers | Primer sequences | GenBank accession | Annealing temp. (℃) | Ref. |
| --- | --- | --- | --- | --- |
| *Gapdh* | F-5’-ATTCTTCCACCTTTGATGCTGG-3’ | NM_017008.4 | 58.91 | 22 |
|  | R-5’-TGCTGTAGCCATATTCATTGTCA-3’ | NM_017008.4 | 58.22 | 23 |
| *CYP7A1* | F-5’-ACCAGGTCTCTGAACTGATCC-3’ | NM_012942.2 | 58.82 | 21 |
|  | R-5’-GAATAGCGAGGTGCGTCTTG-3’ | NM_012942.2 | 59.08 | 20 |
| *FXR* | F-5’-TTCCTCAAGTTCAGCCACAGA-3’ | NM_021745.1 | 59.65 | 21 |
|  | R-5’-GCATCTCGGATACCTCAGTCT-3’ | NM_021745.1 | 58.76 | 21 |
| *PPARγ* | F-5’-CCTTTACCACGGTTGATTTCTC-3’ | NM_001145366.1 | 57.32 | 22 |
|  | R-5’-AGGCTCTACTTTGATCGCACTT-3’ | NM_001145366.1 | 59.77 | 22 |
| *SHP* | F-5’-GCAAGACTGTAGCCTTCCTCA-3’ | NM_057133.1 | 59.72 | 21 |
|  | R-5’-CACTGTTGGGTTCCTCTAGCA-3’ | NM_057133.1 | 59.65 | 21 |
| *Abcg5* | F-5’-CAGACAGGACACCAGAGGATT-3’ | NM_053754.2 | 59.09 | 21 |
|  | R-5’-CTGACGCTGAAGGACACATTC-3’ | NM_053754.2 | 59.27 | 21 |
| *LXRα* | F-5’-AGCGTCCATTCAGAGCAAGT-3’ | NM_031627.2 | 59.68 | 20 |
|  | R-5’-CCACCTCTAGGAAGCAGTCAG-3’ | NM_031627.2 | 59.52 | 21 |
| *FGF15* | F-5’-ACCACCTCCACATCATCTTCAT-3’ | NM_130753.2 | 59.15 | 22 |
|  | R-5’-ACCACCTCCACATCATCTTCAT-3’ | NM_130753.2 | 59.44 | 21 |
| *FGFR4* | F-5’-AGCAAGCACCTTACTGGACAC-3’ | NM_001109904.1 | 60.54 | 21 |
|  | R-5’-CCGAATGCCTCCGATACGA-3’ | NM_001109904.1 | 59.34 | 19 |

**Table S4** The changes in the key lipid parameters in the liver and feces

| **Items(mg/g)^1^** | **Groups^2^** | | | ***P* values^3^** |
| --- | --- | --- | --- | --- |
|  | **Control** | **Model** | **WHHPRO^TM^** |  |
| Hepatic TG | 15.81±0.43 ^c^ | 31.37±0.62 ^a^ | 24.88±0.51 ^b^ | ＜0.0001 |
| Hepatic TC | 11.22±0.34 ^b^ | 19.79±0.57 ^a^ | 11.57±0.40 ^b^ | ＜0.0001 |
| Fecal TG | 18.64±0.66 ^b^ | 26.99±0.60 ^a^ | 25.96±0.67 ^a^ | ＜0.0001 |
| Fecal TC | 19.76±0.43 ^c^ | 29.67±0.36 ^b^ | 39.56±0.49 ^a^ | ＜0.0001 |

^1^ HDL: high-density lipoprotein; LDL: low-density lipoprotein; TG: triglycerides; TC: total cholesterol.

^2^ Results are presented as means ± SEM. Different letters indicate a significant difference.

^3^ *P* values＜0.05 are considered statistically significant.

**Supplementary figures**


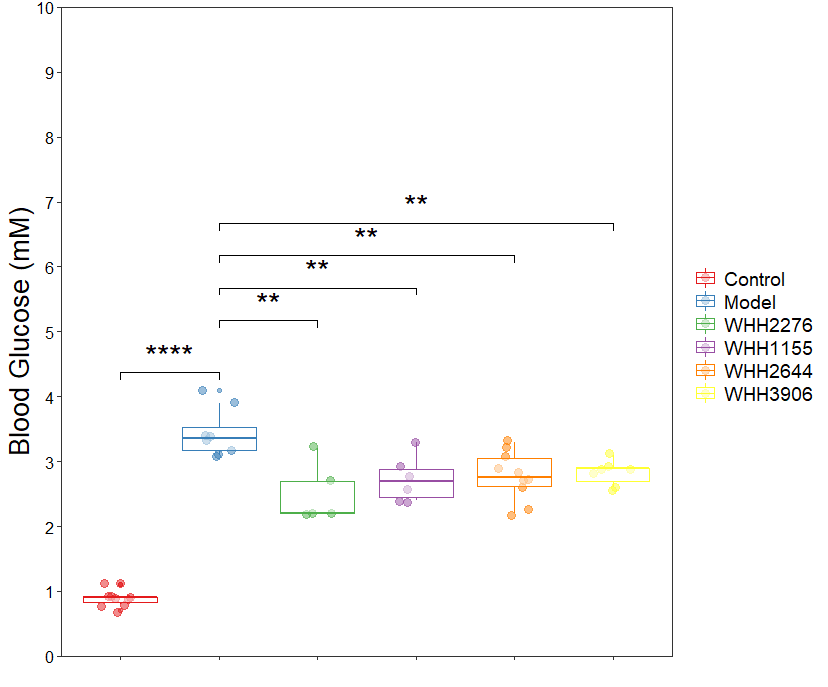


**Figure S1.** Effects of single strains on the levels of blood glucose in zebrafish (n = 10). Data are expressed as medians ± 95% CI. ^*^*P <* 0.05, ^**^*P <* 0.01, ^***^*P <* 0.001 and ^****^*P <* 0.0001. One-way ANOVA with Tukey’s *post hoc* test for all groups.


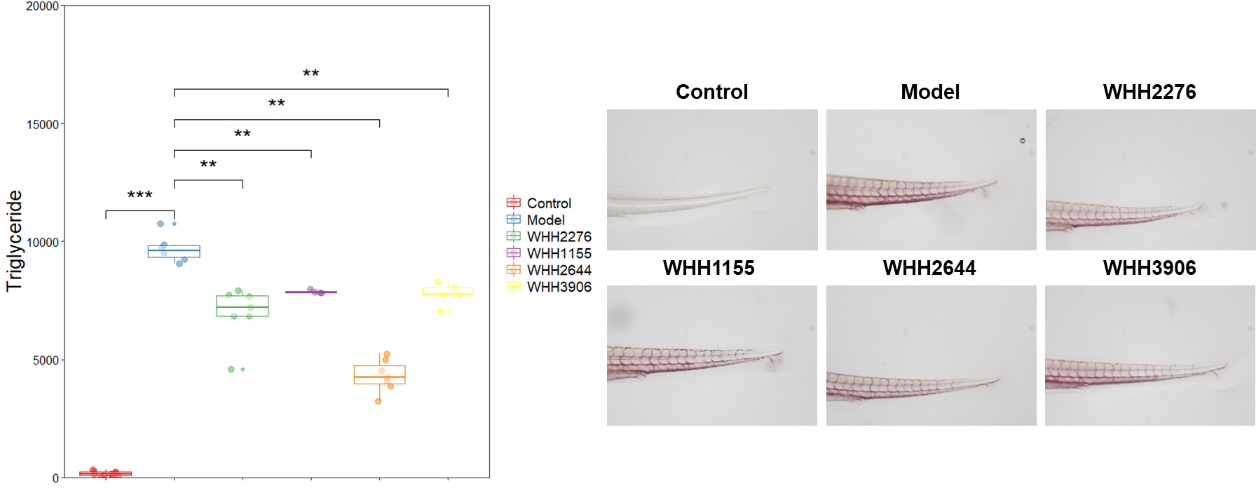


**Figure S2.** Effects of single strains on the levels of triacylglycerol in zebrafish (n = 10). Data are expressed as medians ± 95% CI. ^*^*P <* 0.05, ^**^*P <* 0.01, and ^***^*P <* 0.001. One-way ANOVA with Tukey’s *post hoc* test for all groups.


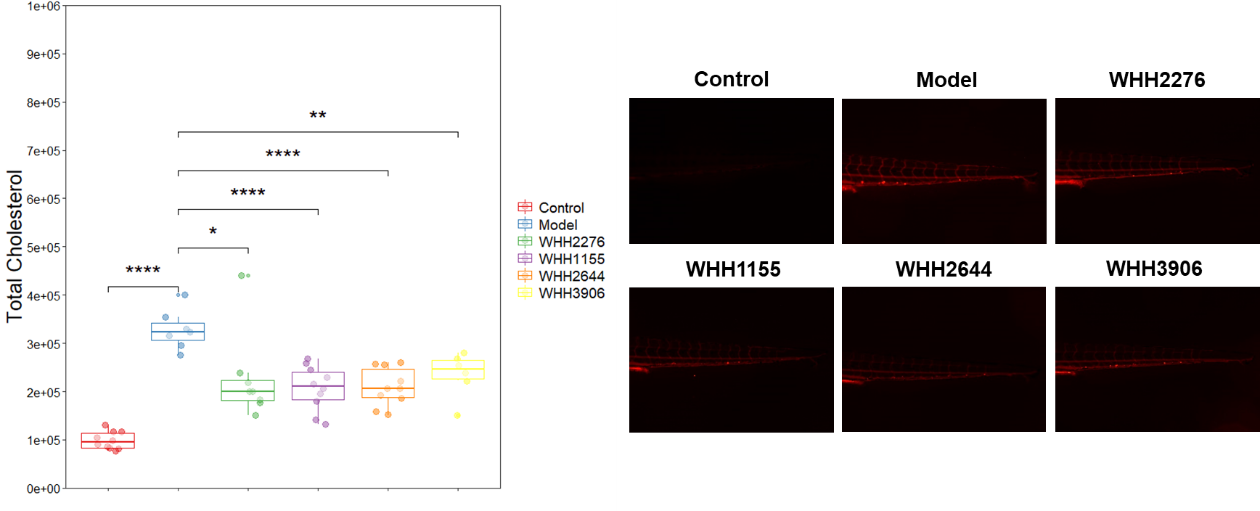


**Figure S3.** Effects of single strains on the levels of total cholesterol in zebrafish (n = 10). Data are expressed as medians ± 95% CI. ^*^*P <* 0.05, ^**^*P <* 0.01, ^***^*P <* 0.001 and ^****^*P <* 0.0001. One-way ANOVA with Tukey’s *post hoc* test for all groups.


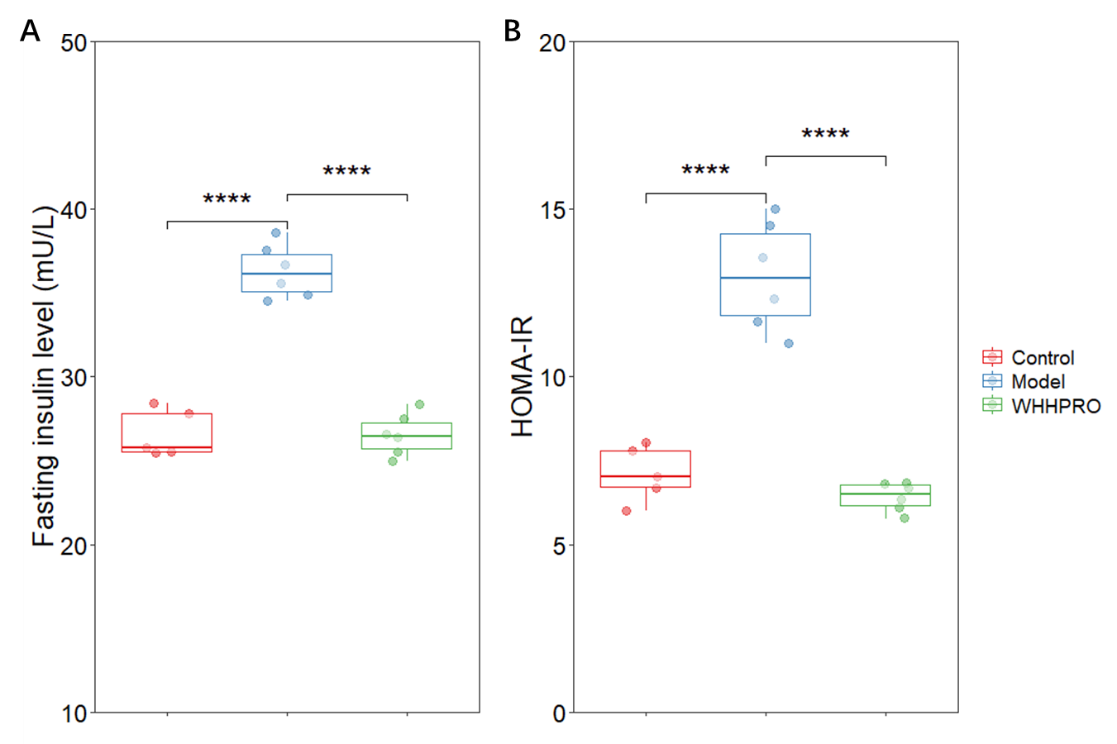


**Figure S4.** Effects of WHHPRO^TM^ on the fasting insulin level (A) and HOMA-IR (B) in rats (n = 5~6). Data are expressed as medians ± 95% CI. ^*^*P <* 0.05, ^**^*P <* 0.01, ^***^*P <* 0.001 and ^****^*P <* 0.0001. One-way ANOVA with Tukey’s *post hoc* test for all groups.
